# Supplementary material for: Optimization of Extraction Solvent and Fast Blue BB Assay for Comparative Analysis of Antioxidant Phenolics from Cucumis melo L
Source: Plants (Basel). 2021 Jul 6;10(7):1379. doi: 10.3390/plants10071379 (PMC8309368; doi:10.3390/plants10071379)
Supplement: Supplementary file 1 [file plants-10-01379-s001.zip › plants-1268942-supplementary.zip/plants-1268942-sup/plants-1268942-Table S1-S2.pdf]

Optimization of Extraction Solvent and Fast Blue BB Assay for Comparative Analysis of Antioxidant Phenolics From *Cucumis melo* L.

Varsha Ravindranath[1,2], Jashbir Singh[1], Guddadarangavvanahally K. Jayaprakasha[1], and Bhimanagouda S. Patil1,2*

1 Vegetable & Fruit Improvement Center, Department of Horticultural Sciences, Texas A&M University, 1500 Research Parkway, Suite A120, College Station, Texas 77845-2119, United States; varsharavindranath@tamu.edu; singh2014@tamu.edu

2 Department of Food Science and Technology, Texas A&M University, College Station, TX, 77845, USA ; b-patil@tamu.edu

*Corresponding author

Bhimanagouda S. Patil

Tel.: +1 979 862 4521; fax: +1 979 862 4522

e-mail: b-patil@tamu.edu

Table S1. Solvents (22 combinations) for optimization of extraction solvents.

SOLVENT ID	SOLVENT COMBINATION
S1	Water
S2	Methanol : water : formic acid 50:45:5
S3	Methanol : water : formic acid 50:48:2
S4	Methanol : water : formic acid 80:15:5
S5	Methanol : water : formic acid 80:18:2
S6	Methanol : water 50:50
S7	Methanol : water 80:20
S8	Methanol
S9	Ethanol : water : formic acid 50:45:5
S10	Ethanol : water : formic acid 50:48:2
S11	Ethanol : water : formic acid 80:15:5
S12	Ethanol : water : formic acid 80:18:2
S13	Ethanol : water 50:50
S14	Ethanol : water 80:20
S15	Ethanol
S16	Acetone : water : formic acid 50:45:5
S17	Acetone : water : formic acid 50:48:2
S18	Acetone : water : formic acid 80:15:5
S19	Acetone : water : formic acid 80:18:2
S20	Acetone : water 50:50
S21	Acetone : water 80:20
S22	Acetone

Sample extracts obtained from 22 solvent combinations were used to evaluate total phenolic content (Folin Ciocalteau assay and optimized Fast Blue assay), and examine antioxidant activities (DPPH and ABTS).

Table S2. Juices used for optimization and validation of the FB assay.

JUICE ID	JUICE SAMPLE
J1	White Grape Juice (Welch's)
J2	White Grape Juice (H-E-B)
J3	Apple Juice (Old Orchard)
J4	Apple Juice (Feeding America)
J5	Pineapple Juice (H-E-B)
J6	Pineapple Juice (Central Market)
J7	Grapefruit Juice (Ocean Spray)
J8	Grapefruit Juice (H-E-B)
J9	Organic Grape Juice (H-E-B)
J10	Concord Grape Juice (Central Market)
J11	Cranberry Juice (Ocean Spray)
J12	Cranberry Juice (Lakewood Organic)
J13	Prune Juice (Sunsweet Amazin)
J14	100% Prune Juice (H-E-B)
J15	Pomegranate Juice (Langers)
J16	Pomegranate Juice (Central Market)

Juice ID and total phenolic contents of the juices are represented in Table 2 of the manuscript.
